# Editorial: Biological and genetic basis of agronomical and seed quality traits in legumes

**DOI:** 10.3389/fpls.2022.1009980

**Published:** 2022-09-29

**Authors:** Jose C. Jimenez-Lopez, Alfonso Clemente, Sergio J. Ochatt, Maria Carlota Vaz Patto, Eric Von Wettberg, Petr Smýkal

**Affiliations:** ^1^Department of Biochemistry, Cell and Molecular Biology of Plants, Estacion Experimental del Zaidin, Spanish National Research Council (CSIC), Granada, Spain; ^2^The UWA Institute of Agriculture and School of Agriculture and Environment, The University of Western Australia, Perth, WA, Australia; ^3^Department of Physiology and Biochemistry of Animal Nutrition, Estacion Experimental del Zaidin, Spanish National Research Council (CSIC), Granada, Spain; ^4^Agroécologie, InstitutAgro Dijon, INRAE, Université de Bourgogne-Franche Comté, Dijon, France; ^5^Instituto de Tecnologia Química e Biológica António Xavier (ITQB NOVA), Universidade Nova de Lisboa, Oeiras, Portugal; ^6^Department of Plant and Soil Science and Gund Institute for the Environment, University of Vermont, Burlington, VT, United States; ^7^Peter the Great St. Petersburg Polytechnic University, St. Petersburg, Russia; ^8^Department of Botany, Faculty of Science, Palacký University, Olomouc, Czechia

**Keywords:** genetic resources, legume breeding, seed traits, food security, sustainable agriculture, global climate change, environmental stresses

Grain legumes present outstanding nutritional and nutraceutical properties while being an economically affordable plant-based protein source for humans that contributes to achieving future food and feed security in the context of an increasing world population. Legumes also provide crucial services to agriculture through their ability to fix atmospheric nitrogen by rhizobial symbiosis. This supplies accessible nitrogen to agroecosystems, increases soil carbon content, stimulates the productivity of subsequent crops by increasing the effective capture, productive use, and recycling of water and nutrients, and helps to control weeds. In this context, genetic improvements have played a pivotal role to increase crop production, from traditional breeding to the most advanced and novel current biotechnological techniques, the application of which will undoubtedly contribute to sustainable agriculture and food security. The Research Topic comprises 29 articles, including 26 Original Research and 3 Review articles covering different aspects focused on agronomic and seed quality traits in legumes. This Editorial summarizes some of the highlights included in the Research Topic.

Serra-Picó et al. investigate the influence of inflorescence architecture on plant traits such as plant shape, morphological diversity, position and number of flowers and fruits produced by the plant, and seed yield. Most legume flowers are produced in secondary inflorescences, and the pea *VEGETATIVE1/FULc* (*VEG1*) gene, and its homologs in other legumes, determine the secondary meristem. In this paper, the authors try to understand the control of this secondary meristem development by identifying downstream genes of *VEG1* as potential regulatory targets of *VEG1*. Two genes were identified whose silencing led to small plants (*PsDAO1*) and plants with very large stubs (*PsHUP54*) and were demonstrated to control the activity of the secondary meristem.

Zhao et al. characterize the common genetic basis underlying seed quality traits in soybean and examine its optimization through selection focused on QTLs useful for breeding new high-yielding soybean varieties with favorable quality characteristics.

Sun et al. identify numerous soybean C2 domain genes that play essential biological functions. The C2 domain genes fell into three distinct groups with diverged gene structures and conserved functional domains. Transcriptional analysis unveiled that genes like *GmC2-58, GmC2-88*, and *GmC2-148* were highly expressed under salt and drought stresses. Moreover, soybean plants with *GmC2-148* transgenic hairy roots increased transcript levels of several abiotic stress-related marker genes, including *COR47, NCDE3, NAC11, WRKY13, DREB2A, MYB84, bZIP44*, and *KIN1* which resulted in enhanced abiotic stress tolerance in soybean. All the results indicated that C2 domain genes are involved in the response to salt and drought stresses.

Desoky et al. investigate nano-silicon application to mitigate the deleterious impacts of drought stress on field crops, an increasing consequence of climate change, particularly in arid regions. Nano-silicon treatment was foliar applied during two seasons of field studies and its influence on crop water productivity (CWP) and the agronomic traits, as well as on physiological and biochemical features were investigated in faba bean (*Vicia faba*). Drought stress significantly decreased gas exchange, water relations, nutrient uptake, flavonoids, and phenolic content. In contrast, drought stress significantly increased oxidative stress (H_2_O_2_ and O^−2^) and enzymatic and non-enzymatic antioxidant activities compared with the well-watered treatment. These results confirm that exogenously applied nano-silicon could be used to improve the CWP and seed and biological yields of faba bean plants under conditions with low water availability in arid environments.

Joe Martin et al. investigate the development of high-folate (Vitamin B9) cultivars of common bean (*Phaseolus vulgaris* L.) and other crops as a tool to improve plant-derived folate of potential use in the human diet. Candidate genes were identified that might be targeted for the development of molecular markers for selecting *P. vulgaris* cultivars with improved seed folate content.

Das et al. describe how up-to-date research has advanced in multiple features of grass pea thanks to the significant repositories of *Lathyrus* germplasm with a wide range of agro-morphological traits as well as a low β-ODAP content available across countries. They also discuss the genetic enhancement of grass peas to make this food safe for human consumption and increase climate resilience.

Gunjača et al. phenotype 174 common bean (*Phaseolus vulgaris* L.) accessions through the seed content of N, P, K, Ca, Mg, Fe, Zn, and Mn, and genotype them using 6,311 high-quality DArTseq-derived SNP markers. Then, 31 quantitative trait nucleotides (QTN) associated with seed nutrient content were identified by GWAS: 22 on chromosomes Pv01, Pv02, Pv03, Pv05, Pv07, Pv08, and Pv10 for nitrogen; five (4 on chromosome Pv07, and one on Pv08) for phosphorus, a single significant QTN for calcium on chromosome Pv09, one for magnesium on Pv08, and two QTNs for seed zinc content on Pv06. No QTNs for potassium, iron, or manganese content were found. These results highlight the relevance of GWAS in unraveling the genetic makeup of seed nutritional traits in common beans.

Roorkiwal et al. survey the molecular mechanisms of nuclear acquisition, transport, and metabolism aimed at supporting a biofortification strategy for grain legume crops. They evoke the reasons for malnutrition globally and the role of legume crops in eradicating it. They examine nutrient-wise the mechanisms of mineral acquisition and transport, and the metabolic pathways for vitamins (β-carotene, folate, vitamin E) and anti-nutritional factors (phytic acid, raffinose). They plunge into the potential interventions through biofortification (agronomic, genetic breeding, genome engineering), and continued with a review of the genomic approaches to nutritional breeding (genetic variations of micronutrients in legumes and identification of QTLs/genes to unveil the genetic architecture of nutrient accumulation). Their survey is completed by a prospective analysis of the role of genomics in nutritional breeding.

Crosta et al. examine genotype x environment interaction (GEI), genetically based trade-offs, and polygenic control for crude protein content and grain yield of peas to assess genomic selection (GS) efficiency vs. phenotypic selection (PS). They analyze 306 lines through genotyping-by-sequencing, and for grain yield and protein content in three autumn-sown environments in Italy. Compared with GEI, purely genetic effects were >2-fold larger for protein content, but >2-fold smaller for grain and protein yield, while grain yield and protein content exhibited no inverse genetic correlation. GWAS revealed a polygenic control for grain yield and protein content, with limited trait variation accounted for by individual loci. GS predictive ability for individual RIL populations holds promise for the simultaneous improvement of protein content, and grain and protein yield.

Dadu et al. examine the genetic basis of *Ascochyta blight* resistance in lentils, a significant disease that impacts production around the globe. Using a wild/cultivated cross, they found a major QTL with three component parts on the fifth chromosome of lentils, explaining nearly 10% of the variation in resistance and containing several putative candidate genes. The resistance QTL should prove useful for introgressing alleles conferring resistance into elite lentil cultivars.

Rane et al. use top and side view imaging to model the growth rate and biomass differences in response to water limitation in mungbean (*Vigna radiata* L. Wilczek), a short cycle legume with considerable capacity to grow in agriculturally marginal soils. The phenomic approach they use can help lower costs in characterizing germplasm, and better reflects low input agricultural conditions common across much of the world's semi-arid tropics.

To better understand genetic variation underlying productivity and quality traits in peanut (*Arachis hypogaea* L.), an allotetraploid crop that has trailed some others due to low variability in elite germplasm, Jadhav et al. characterize a cross between an elite variety and an EMS-treated line. In this cross, with 700 markers, 47 main-effect QTLs for the productivity and oil quality traits explaining between 10 and 50% of phenotypic variation were uncovered, several of which showed epistatic interactions. They identify potential candidate loci for future molecular breeding in groundnut.

Atieno et al. map salinity tolerance under hydroponic and field conditions in Western Australia, using a RIL population of two elite desi chickpea (*Cicer arietinum* L.) parents adapted to Australian agroecosystems. The researchers find six QTLs across three chromosomes that mapped to salinity tolerance *per se*, as well as other QTLs related to phenology and aspects of vigor such as seed and leaf size. Fine mapping of these QTLs will yield useful alleles for improving the salt tolerance of chickpeas.

Guiguitant et al. examine trait variation, such as leaf nitrogen and leaf area, in 30 cultivars of 10 legume species to understand axes of trait combinations that could be selected to adapt leguminous crops to different climates and agroecological conditions. The authors find significant trade-offs between traits across climatic conditions, consistent with an economic spectrum of trait values, with some trait combinations most suited to different agronomic situations. The trait-based approach could facilitate more rapid adaptation of leguminous crops to novel agricultural settings.

Peas (*Pisum sativum* L.), a cool season legume, suffer from heat stress. Lamichaney et al. examine the variation in responses to heat stress, imposed by late planting in India, in 150 pea cultivars and find substantial variation in a range of traits that impact tolerance. Four accessions are identified as possessing superior heat tolerance and are suitable for rapidly warming climatic conditions being experienced in India's pea production regions.

The *Catharanthus roseus* RLK1-like (CrRLK1L) protein kinase family is involved in a range of abiotic stress responses in plants. Wang Z.-Q. et al. examine the expression of 38 *CrRLK1L* genes in soybean (*Glycine max* L.), finding that one member of the family, *GmCrRLK1L20*, is upregulated in response to drought and salt stress. This gene could be a target of manipulation for improving soybean stress tolerance.

Yang et al. map genetic variation in forage quality traits, such as protein content and fiber composition, in a biparental population of alfalfa (*Medicago sativa* L.). Across several different traits, the authors find 83 QTLs, several of which interacted with several traits or had epistatic interactions with each other. In over 80% of the co-localized QTLs, alleles had opposite effects on protein content and fiber composition, suggesting trade-offs in these characteristics. The identified QTLs provide several potential targets for marker-assisted selection.

Chen Z.-F. et al. provide novel insight into the detection and function of *GmPLC* (Phospholipase C) genes in soybean plants subjected to drought and salinity through a Genome-Wide Analysis of the PLC proteins family that catalyze phospholipids hydrolysis. They identified 24 specific PLC genes which mapped to 10 of the 20 soybean chromosomes, grouping them into phosphatidylinositol-specific PLC (*GmPI-PLC*) and phosphatidylcholine-hydrolyzing PLC genes. Selecting *GmPI-PLC7*, they show that compared to transgenic empty vector controls, *GmPI-PLC7* (OE) overexpressors conferred higher drought and salt tolerance, opposite to the *GmPI-PLC7*-RNAi (RNAi) lines. Under such stresses, the OE exhibited higher amounts of chlorophyll, oxygen free radical, H_2_O_2_, and NADH oxidase than those in non-stressed plants. *GmPI-PLC7* may improve stress tolerance in soybean through ABA signaling and the SOS-related calcium-signaling pathway.

Different environmental and production pressures (pests and diseases, drought, and differing grazing management, as well as introduced legislation restricting farming practices) have changed the targets of the legume forage *Trifolium* spp. breeding across time. Egan et al. review the pre-breeding and breeding programs history of white, red, and minor clover sp. in New Zealand, with a particular focus on the role of gene banks as a source of interesting materials. They further focus on the proper characterization of the materials prior to use, a task facilitated by recent biotechnological and molecular advances.

Fast cooking is an important trait influencing common bean consumers not only from the economic (energy and time consumption) but also from the nutritional (minerals and proteins retention) point of view. To support quality common bean breeding, Diaz et al. study the genetic architecture related to the cooking time and water absorption using linkage, association, and use a combination of both mapping approaches and genomic prediction applied to all the studied populations. Different QTLs for the Andean and Mesoamerican gene pools were located in distinct regions of the genome, suggesting differential genetic control in each of the pools for the traits of interest. Genomic prediction accuracies varied dependent on the population under study, but the generated phenotypic characterization data set will facilitate cooking time being incorporated into breeding programs.

Most Andean common bean cultivars are photoperiod sensitive (in a temperature-dependent way), while Mesoamerican and determinate cultivars include a high portion of day-neutral lines. To support common bean breeding for different climatic and planting time conditions, Gonzalez et al. study the genes involved in flowering time and photoperiod response using a multi-environment (long and short day) QTL mapping approach within the Andean gene pool. Two novel major loci were detected on chromosomes 4 and 9 controlling flowering under long and short day lengths respectively, showing complex epistatic and environment interactions.

Secondary metabolites are used by plants as defensive agents to survive threats derived from abiotic and biotic stresses. In soybean leaves, coumestrol functions as a phytoalexin and increases its levels as the plant ages and also upon insect attack. Mun et al. report the accumulation of coumestrol during leaf maturity or senescence in soybean when applied with several phytohormones including salicylic acid, methyl jasmonate, and ethephon alone or in several combinations. These findings might be exploited as a novel strategy to increase coumestrol levels in soybean leaves for further industrial exploitation.

Venugopalan et al. investigate the influence of time of sowing and a foliar spray of micronutrients (boron, iron, and zinc, either alone or in combination) to diminish the effect of moisture and heat stress on lentils (*Lens culinaris* Medik.) in the subtropical region. Crop growth rate and biomass were significantly affected by the time of sowing and the treatment of a foliar spray of micronutrients when compared to soil application. This study supports the importance of the appropriate date of sowing and foliar spray treatment to increase the sustainability of lentil production under abiotic stresses such as high temperature and moisture that could result in higher yield.

The yield of peanut crops is limited by water deficits. Therefore, the development of drought-tolerant varieties adapted to drought stress is a major aim in peanut breeding programmes. Wang X. et al. examine different peanut drought-tolerant and drought-susceptible genotypes to identify drought-induced genes expressed under drought stress conditions. The reported data reveals the involvement of a complex network of phytohormones in the regulatory mechanisms of peanut drought tolerance.

Wang N. et al. study the plant responses to the combined effect of drought and defoliation treatment in two legume species (*Robinia pseudoacacia* and *Amorpha fruticosa*). Data obtained demonstrate that defoliation could alleviate the effect of low water availability in large seedlings. These results will be helpful to gain knowledge of forest dynamics under climate change conditions and support further studies in terms of vegetation restoration.

Seck et al. study the genetic basis of root system architecture in soybean through GWAS analysis. They characterize twelve root traits in a panel of 137 early maturing soybean lines (Canadian soybean core collection) using rhizoboxes and two-dimensional imaging. In total, 10 quantitative trait locus (QTL) regions were detected for root total length and primary root diameter, which could be used to develop climate-resilient soybean cultivars.

Bassett et al. identify a QTL that can be used to develop molecular markers to improve seed quality traits in dry bean varieties. The authors used a recombinant inbred line population that was developed from a cross between Ervilha (Manteca) and PI527538 (Njano), yellow dry beans with contrasting cooking time and sensory attributes. This study detects QTLs for water uptake, cooking time, sensory attribute intensities, color, and a non-darkening seed-coat postharvest.

Chen Z. et al. study the impact of moderate heat stress on the model legume *Medicago truncatula*, applied at flowering onwards, to the seed development and maturation, particularly on seed weight and germination capacity, and the identification of quality traits and regulatory genes to control these seed features. In particular, *MtMIEL1*, a RING-type zinc finger family gene was shown to be highly associated with the germination speed of heat-stressed seeds. The conservancy of gene function is demonstrated by a loss-of-function analysis of the *Arabidopsis MIEL1* ortholog, supporting its role as a regulator of the germination plasticity of seeds in response to heat stress.

Carlson-Nilsson et al. investigate the specific factors that cultivated crops need to adapt to in adverse and extreme climate conditions, such as low temperatures, long days, and a short growing season. They further identify suitable pea genetic resources for future cultivation and breeding in the Arctic region. The data showed that light conditions related to a very long photoperiod partly compensated for the lack of accumulated temperature in the far north. A critical factor for cultivation in the Arctic is the use of cultivars with rapid flowering and maturation times combined with an early sowing. Altogether, the results identify pea genetic resources available for breeding or immediate cultivation, aiding in the northward expansion of pea cultivation.

## Author contributions

JCJ-L, AC, SO, MV, EV, and PS have written, reviewed, and edited the original draft. All authors have approved the final manuscript.

## Funding

JCJ-L acknowledges financial support from the Spanish Government grant ref. RYC-2014-16536 (Ramon y Cajal research program of MINECO), and by the European Research Program MARIE CURIE (FP7-PEOPLE-2011-IOF) grant ref. number PIOF-GA-2011-301550. AC acknowledges the financial support of AGL2017-83772-R (AEI/FEDER, UE) and P20_00242 (PAIDI 2020) funded by the Spanish Ministry of Science and Innovation, and Junta de Andalucía, respectively. MV acknowledges the support of Fundação para a Ciência e Tecnologia, Portugal through the R&D research Unit GREEN-IT – Bioresources for Sustainability (UID/04551/2020, UIDP/04551/2020), and the LS4FUTURE Associated Laboratory (LA/P/0087/2020). EV's contribution to this work was supported by the Ministry of Science and Higher Education of the Russian Federation as part of the World-class Research Center program: Advanced Digital Technologies (contract No. 075-15-2022-311 dated 20.04.2022).

## Conflict of interest

The authors declare that the research was conducted in the absence of any commercial or financial relationships that could be construed as a potential conflict of interest.

